# Online–Offline Social Interaction, Interpersonal Well-Being, and Age Cohort Resilience Among Chinese Adolescents

**DOI:** 10.3390/bs16091537

**Published:** 2026-09-01

**Authors:** Mengru Sun

**Affiliations:** 1 College of Media and International Culture, Zhejiang University, Hangzhou 310058, China; mengrusun@zju.edu.cn; 2 International Communication Institute, Zhejiang University, Hangzhou 310058, China

**Keywords:** online communication, offline communication, quality of interpersonal relationships, adolescents, age cohort

## Abstract

Research has documented the various effects of internet use on adolescent well-being. However, few studies have integrated the Displacement Hypothesis and the Stimulation Hypothesis to compare the effects of online and offline communication on the quality of interpersonal relationships among adolescents. Moreover, limited research has examined the role of age cohort in these relationships. The present study addresses these gaps by investigating the age-related differences in these associations. Specifically, we examined the influence of both online and offline communication on the quality of adolescents’ interpersonal relationships and explored the moderating role of age cohort. Data were drawn from the China Family Panel Studies, a nationally representative survey. A cross-sectional sample of 1486 adolescents aged 10 to 15 years (54.4% boys and 45.6% girls) was analyzed using correlation, hierarchical regression, and bootstrap-based mediation analyses. The results indicated that adolescents who engaged in more online communication also tended to engage in more offline communication. Furthermore, both online and offline communication are jointly related to the quality of interpersonal relationships among adolescents. Importantly, age cohort was found to moderate the effects of both online and offline communication on the quality of interpersonal relationships. Theoretical and practical implications are discussed.

## 1. Introduction

The digital age has witnessed tremendous growth in internet users worldwide, with adolescents constituting one of the most active user groups. In the United States, 95% of adolescents have access to a smartphone and 46% report being online almost constantly (Pew Research Center, 2023). In China, the internet penetration rate among minors has reached 97.3%, with the number of underage internet users reaching 196 million and the trend toward first internet use at increasingly younger ages persisting (China Internet Network Information Center, 2024). As the daily time spent online by adolescents continues to rise, online interaction has become an integral part of their lives, playing a significant role in shaping their interpersonal relationships. For instance, more than 90% of American teens now use at least one major social media platform, with YouTube being the most popular (93%), followed by TikTok (63%), Snapchat (60%), and Instagram (59%) (Pew Research Center, 2023). Similarly, online chatting and social interaction remain among the most common online activities of Chinese underage internet users, for whom the internet has become an important window for understanding the world and maintaining peer relationships.

Research has documented the effects of internet use on adolescent well-being across multiple dimensions, including life satisfaction (Grieve et al., 2013), loneliness (Bonetti et al., 2010; Zhen et al., 2019), depression (Tandoc et al., 2015), and anxiety (Caplan, 2002). However, theoretical approaches explaining these phenomena have yielded inconsistent assumptions and findings. While some scholars contend that excessive internet use may cause both physical and mental health problems among adolescents (Brenner, 1997; Griffiths, 2000; Young, 1996), other studies have found no significant association between internet use and well-being (Gross et al., 2002; Feng et al., 2025). More recent large-scale studies likewise indicate that the associations between digital technology use and adolescent well-being are heterogeneous and typically small, varying considerably from one adolescent to another (Beyens et al., 2020; Dienlin & Johannes, 2020). Furthermore, a substantial body of research suggests that online communication is positively related to interpersonal relationship quality (e.g., Valkenburg & Peter, 2007b; Valkenburg et al., 2022). In general, studies examining the effect of online communication on adolescent friendships have centered on two well-known theoretical frameworks: the Displacement Hypothesis and the Stimulation Hypothesis. Although these two hypotheses advance opposing propositions, they share a common assumption that online communication affects offline communication and, in turn, the quality of interpersonal relationships. Notably, the quality of interpersonal relationships is positively associated with adolescent well-being, encompassing psychological outcomes such as lower depression and anxiety and greater life satisfaction (Grieve et al., 2013), as well as behavioral outcomes such as problematic mobile phone use (Zhen et al., 2019). In the Chinese context, recent evidence also indicates that the intensity of mobile social media fatigue is negatively associated with the quality of friendships among youth (Sun & Li, 2026).

Despite the substantial body of research guided by these two perspectives, few studies have integrated them by directly comparing the effects of online and offline communication on the quality of interpersonal relationships among adolescents. Additionally, limited research has investigated the role of individual differences in these relationships, particularly from the perspective of age cohort. Therefore, the present study aims to fill these gaps by examining age-related differences in these associations. Specifically, this study investigates the influence of both online and offline communication on the quality of adolescents’ interpersonal relationships and explores the moderating role of age cohort in these relationships. This study not only articulates the Displacement Hypothesis and the Stimulation Hypothesis but also advances our understanding of these two theoretical frameworks by proposing a moderating effect of age cohort. In doing so, the present study moves beyond testing the Displacement and Stimulation Hypotheses in isolation by embedding their competing predictions in a single mediation model and by identifying age cohort as a developmental boundary condition of these processes.

## 2. Literature Review

### 2.1. Online Communication and Interpersonal Relationships

Although adolescent internet use has attracted worldwide attention, the association between internet use and well-being remains contingent on various factors. Some studies have found no significant association between internet use and relational outcomes such as closeness and satisfaction (Hu & Yan, 2025; Gross et al., 2002; Feng et al., 2025). Conversely, other research has indicated that excessive time spent online may lead to the neglect of family and social life, alienation from friends, and deterioration of interpersonal relationships (Demetrovics et al., 2008; Kuss et al., 2014; Ryding & Kaye, 2018). Furthermore, internet addiction may give rise to both physical and psychological problems (Brenner, 1997; Griffiths, 2000; Young, 1996), including problematic internet use behaviors, disrupted sleep (Brenner, 1997), impaired academic performance, anxiety, distress, and depression (Caplan, 2002; Davis, 2001; Demetrovics et al., 2008).

On the other hand, there is a growing consensus that internet use for social interaction purposes—that is, online communication—is positively associated with adolescent well-being. Scholars in this line of research argue that using the internet for relationship maintenance can exert beneficial psychological effects by temporarily reducing stress and anxiety (Leung, 2006). Empirical evidence has revealed that online communication is associated with lower depression and anxiety and greater life satisfaction (Grieve et al., 2013). More specifically, for adolescents who perceive low friendship quality, internet use for communication purposes is associated with lower levels of depression, lending support to the social compensation effect of online communication on depression. More recent work corroborates these benefits while emphasizing heterogeneity: the effects of social media use on well-being differ substantially across adolescents and depend on how young people engage with these platforms (Beyens et al., 2020; Weinstein, 2018).

Furthermore, a growing body of research has focused on the association between online communication and the quality of interpersonal relationships. Adolescents perceive their online social interactions as similar to their offline communication with friends, discussing fairly ordinary yet intimate topics through instant messaging, email, online forums, and social media (Feng et al., 2025). Moreover, online interaction has been perceived as high in quality by adolescents (Hu & Yan, 2025). These findings suggest that internet use for social interaction can positively influence various types of interpersonal relationships, including peer, parent–child, teacher–student, online–friend, and romantic relationships (Lai & Gwung, 2013).

Research has also demonstrated that online communication is positively related to the closeness of friendships (Moorman & Bowker, 2011; Angelini et al., 2026). Scholars suggest that social media use (e.g., Facebook) may provide opportunities to develop and maintain social connectedness in the online environment, offering an alternative social outlet to fulfill critical needs for social interaction, self-disclosure, and identity exploration (Grieve et al., 2013; Bonetti et al., 2010). Importantly, this beneficial effect was observed only among those who primarily communicated online with existing friends, rather than those who mainly interacted with strangers (Angelini et al., 2026). Recent evidence further indicates that such benefits are more likely when adolescents actively communicate with existing friends than when they passively browse others’ content (Seidman et al., 2025; Verduyn et al., 2017).

### 2.2. The Hypotheses of the Effects of Online Communication

Two well-known yet opposing explanatory frameworks have been proposed to account for the effects of online communication on adolescent well-being: the Displacement Hypothesis and the Stimulation Hypothesis (Mýlek et al., 2024). These hypotheses have been tested across various contexts, including online self-disclosure, frequency of online communication, social compensation motives, and different types of media such as instant messaging, cell phones, and social networking sites (Kwak et al., 2014; McDonald et al., 2026; Valkenburg & Peter, 2008). According to the Displacement Hypothesis, online communication impairs adolescent well-being by substituting time devoted to offline interactions with established friends and consequently degrading friendship quality. Conversely, the Stimulation Hypothesis suggests that online communication fosters well-being by reinforcing adolescents’ existing friendships and elevating their relational quality.

Context-bound empirical results have validated each of these two competing hypotheses under different circumstances. Scholars have underscored that face-to-face interaction remains adolescents’ predominant social practice despite the deep integration of digital media into youth social life (Baym et al., 2004). Compared with online friendships, offline friendships are characterized by greater interdependence, broader and deeper exchanges, code-switching, mutual comprehension, commitment, and network convergence (Chan & Cheng, 2004). Still, relational quality increases alongside relationship length for both friendship types, and their distinguishing disparities attenuate over time (Chan & Cheng, 2004). Moreover, a longitudinal study found that while online and offline relationships are strongly and positively correlated, most adolescents do reduce their offline social interactions when their use of social networking sites is restricted (Li & Mora Villarrubia, 2018). Online communication may function as an extension of offline interaction, and some users tend to rely on social media for interpersonal communication more than on face-to-face interaction (Kujath, 2011).

### 2.3. Age Cohort and Interpersonal Relationships

Two key factors suggest that adolescents may increasingly rely on online communication relative to offline communication as they mature, namely, psychosocial developmental needs for intimacy and evolving capacities as relationship partners. On the one hand, adolescence is a life stage characterized by numerous biological, cognitive, and social changes. As newer interactive media have become an integral part of adolescents’ lives, they constitute an important social context that provides youth with opportunities to explore the developmental challenges they face (Cenameri, 2013). Several theoretical frameworks have identified key psychosocial developmental tasks during adolescence. For example, Hill and Lynch (1983) proposed that adolescent behavior is best understood in terms of the developmental tasks of identity, autonomy, intimacy, and sexuality. Similarly, Steinberg et al. (2008) identified three primary developmental tasks: the development of a firm sense of self or self-identity, the development of intimacy, and the development of sexual psychology. Intimacy is widely recognized as a central developmental task, requiring adolescents to acquire the ability to establish, maintain, and, when necessary, terminate close and meaningful relationships. Concurrently, the distinctive advantages of online communication, such as concealment, exploitability, and processability, attract adolescents to engage in it. Research suggests that internet use allows adolescents to fulfill critical needs for social interaction, self-disclosure, and identity exploration (Bonetti et al., 2010). Online communication has enhanced adolescents’ control over self-presentation and self-disclosure, which is likely to promote their social development. In several respects, online communication parallels traditional offline social interaction (Brown, 1999): it occurs largely in private settings (e.g., email and instant messages) with friends who are also part of participants’ daily offline lives, such as school. Furthermore, online communication is primarily devoted to ordinary yet intimate topics (e.g., friends and gossip) and is motivated by a desire for companionship (Gross et al., 2002).

On the other hand, contemporary scholarship acknowledges that adolescent relational capabilities and the characteristics of their social connections vary across developmental stages. While family ties retain considerable significance throughout adolescence, young people tend to allocate growing amounts of time to interactions beyond the household. These non-family relationships fulfill numerous social functions that were once predominantly fulfilled by family bonds in childhood (W. A. Collins & Laursen, 2006). Multiple empirical investigations indicate that friends engage in internet-mediated communication with each other more frequently than family members do (Chen et al., 2002; Dimmick et al., 2000; Wellman & Gulia, 1999).

Taken together, online communication and closeness to friends tend to increase with age, suggesting that as adolescents mature, online communication may assume a more prominent role in their interpersonal relationships, while the converse may hold for offline communication. Existing scholarship characterizes adolescents’ vulnerability to peer-group pressure as following an inverted-U developmental trajectory: susceptibility rises in early adolescence, reaches its peak at approximately 14 years old, and subsequently decreases (Brown, 1990; Krosnick & Judd, 1982; Steinberg & Silverberg, 1986). Similarly, social anxiety has been found to be curvilinearly related to age, with higher levels observed in early and middle adolescence than in preadolescence and late adolescence (Inderbitzen-Nolan & Walters, 2000). The relationship between age and the perceived value of the internet for intimate self-disclosure has also been found to be curvilinear, with 15-year-olds exhibiting the highest levels of online self-disclosure (Angelini et al., 2026). These developmental shifts provide the theoretical rationale for expecting age cohort to moderate not only the direct associations of online and offline communication with relationship quality but also the indirect association through offline communication: as adolescents grow older, their increasing autonomy, peer orientation, and digital competence render online communication a progressively more central channel for building closeness, whereas the relative importance of activity-based offline interaction gradually declines (Weinstein, 2018).

### 2.4. The Present Study

Based on the literature reviewed above, we expect to observe a positive association between adolescents’ online communication and their offline communication. Furthermore, both online and offline communication are expected to be positively related to the quality of interpersonal relationships among adolescents. Finally, we anticipate that older adolescents will place greater importance on online communication in relation to their interpersonal relationship quality compared with younger adolescents, whereas the opposite pattern is expected for offline communication.

The psychosocial and cognitive development of adolescents is best understood when divided into four periods: preadolescence (9–10 years), early adolescence (11–14 years), middle adolescence (15–17 years), and late adolescence (18–21 years). The first three periods have received considerable scholarly attention and correspond to the age range of the present sample. Moreover, given that the distinctions among these three stages are not always clear-cut and the age range of participants in the present study spanned 10 to 15 years, we adapted the age categories into three cohorts: preadolescence (primary school students), early adolescence (middle school students), and middle adolescence (high school and technical school students).

**Figure 1:**
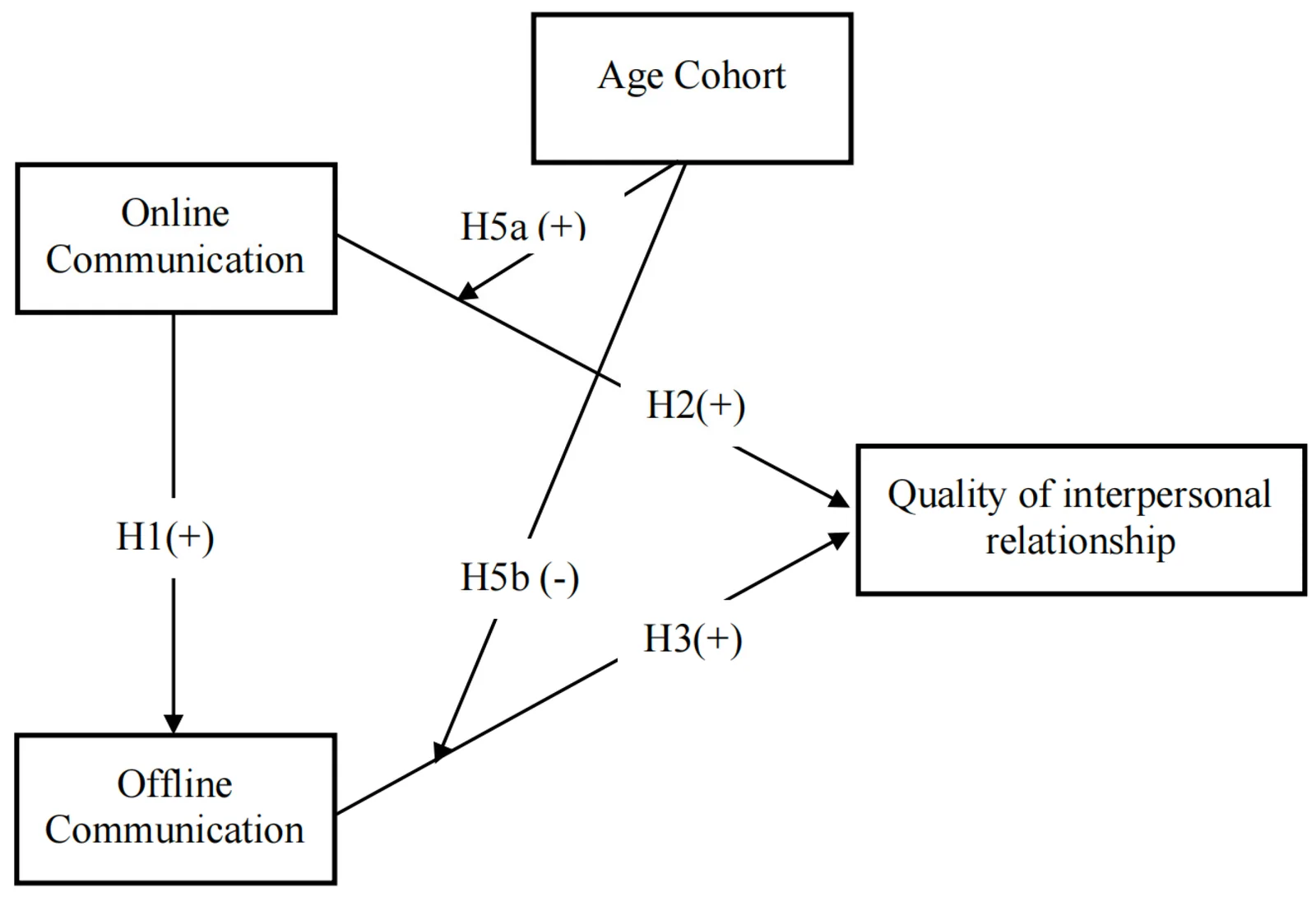
The hypothesis model in the present study.

Regarding the moderating effects on the relationships between online (offline) communication and the quality of interpersonal relationships, we propose the following hypotheses (see Figure 1):

**Hypothesis** **1.** *Online communication is positively related to offline communication among adolescents.*

**Hypothesis** **2.** *Online communication is positively related to the quality of interpersonal relationships among adolescents.*

**Hypothesis** **3.** *Offline communication is positively related to the quality of interpersonal relationships among adolescents.*

**Hypothesis** **4.** *Offline communication mediates the relationship between online communication and the quality of interpersonal relationships among adolescents.*

**Hypothesis** **5a.** *Older adolescents regard online communication as more important in predicting the quality of their interpersonal relationships than do younger adolescents.*

**Hypothesis** **5b.** *Older adolescents regard offline communication as less important in predicting the quality of their interpersonal relationships than do younger adolescents.*

Figure 1. The hypothesized model: the mediating role of offline communication and the moderating role of age cohort.

## 3. Methods

### 3.1. Data

The China Family Panel Studies (CFPS) is a nationally representative, comprehensive social tracking survey designed to reflect changes in China’s society, economy, population, education, and health by collecting longitudinal data at the individual, family, and community levels. The survey provides a valuable data resource for academic research and public policy analysis. The target sample size of the CFPS was 16,000 households, encompassing respondents from 25 provinces, municipalities, and autonomous regions across China, along with all family members within the sampled households (Xie & Lu, 2015). The CFPS defines individuals under the age of 16 as adolescents and those aged 16 and above as adults. The recent wave of the CFPS in 2018 included 2496 adolescent cases. Rather than exploiting the longitudinal structure of the database, the present study used cross-sectional data from the 2018 wave. In the CFPS, an adolescent case refers to an individual under the age of 16 who completed the child questionnaire within a sampled household. Adolescent respondents aged 10 to 15 years provided the data for the present study. The inclusion criteria were as follows: (1) Data from the self-administered individual section was extracted from five components of the full CFPS questionnaire and (2) participants answered a self-report item measuring their current educational stage. Only those selecting options of primary school, junior high school, and senior high school/secondary specialized school/technical school/vocational high school. The exclusion criteria were as follows: (1) respondents who had never used the internet; and (2) respondents with incomplete responses on the study variables. Following this selection process, a final analytical sample of 1486 adolescent cases was retained.

### 3.2. Participants

After data screening based on the objectives of the present study, the sample consisted of 1486 adolescents (see Table 1 for details) between 10 and 15 years of age (M = 12.85, SD = 1.61). The majority of participants were boys (54.4%; n = 809), with 45.6% being girls (n = 677). A slightly larger proportion of adolescents resided in rural areas (52.3%, n = 765) compared with urban areas (47.7%, n = 699). The average level of adolescents’ monthly income (pocket money received from any household member) was 80.59 RMB (SD = 134.54). Only 23 adolescents (1.5%) reported being in a romantic relationship at the time of the survey, indicating that romantic involvement was rare in this sample. In mainland China, romantic relationships among adolescents under the age of 18 are commonly labeled “early love,” a term that carries negative connotations rooted in prevailing social norms and reflects a policy and cultural context that expects adolescents to prioritize academic pursuits over romantic involvement during their teenage years. In the CFPS, informed consent was obtained from all participants, and for adolescent participants, informed consent was obtained from their parents or legal guardians.

### 3.3. Measures

Offline communication. Offline communication was assessed using five indicators adapted from Gross’s (2004) measurement of daily after-school social activities. Four items were measured by asking adolescent participants how often they went out with friends “for dinner, to a disco, to karaoke, or to play in a cybercafe.” The response categories were “Almost daily,” “Several times per week,” “2–3 times per month,” “Once per month,” “Less than once per month,” and “Never”. The fifth item captured both the intensity and variety of social interactions by counting the number of listed activities that the adolescent engaged in at least once per month, ranging from “no activities” to “all four activities”. These items were averaged (M = 0.51, SD = 0.51, Cronbach’s alpha = 0.614), with higher scores indicating a higher level of offline communication. These indicators capture the frequency and variety of adolescents’ face-to-face social activities with friends in their daily after-school lives and thus serve as behavioral indicators of offline peer interaction (Feng et al., 2025).

Online communication. Online communication was assessed using a measure adapted from Kujath’s (2011) social media usage scale. Two indicators were drawn from the survey. The first item asked how frequently the adolescent used the internet for social interaction (e.g., posting on Weibo, sending instant messages). The available response categories were “Almost daily,” “Between 3 and 4 days per week,” “Between 1 and 2 days per week,” “Between 2 and 3 times per month,” “Once per month,” “Less than once in a month,” and “Never.” The second indicator assessed the subjective importance of online social relationships, with adolescents rating how important it was to socialize while online on a scale from 1 to 5, with higher values indicating greater importance. These two items were averaged (M = 3.02, SD = 1.26, Pearson r = 0.368), with higher scores indicating a higher level of online communication. The rationale for averaging these two items is that they capture two complementary facets of adolescents’ online communication involvement—the behavioral frequency of online social interaction and the subjective importance attached to it—consistent with prior research that operationalizes online communication by combining usage frequency with the significance users attach to their online social relationships (Pourasadi et al., 2026; Kujath, 2011).

Quality of interpersonal relationships. The quality of interpersonal relationships was assessed by asking adolescents to rate their interpersonal relationships on a single item ranging from 0 to 10, with higher scores indicating higher perceived relationship quality (M = 7.13, SD = 2.01).

Age cohort. Participants were classified into three age cohorts: preadolescence (primary school students; n = 680, 46.4%), early adolescence (middle school students; n = 750, 51.2%), and middle adolescence (high school and college students; n = 36, 2.5%). Primary school students were coded as “1” (the youngest group), middle school students as “2,” and high school and college students as “3” (the oldest group). The mean of the age cohort variable was 1.56 (SD = 0.54).

### 3.4. Sociodemographic Measures

A set of sociodemographic variables, primarily coded as dummy variables, were included as control variables to account for potential confounding effects. Age was treated as a continuous variable. Gender was coded as 0 = “female” and 1 = “male.” School location was a dichotomous variable (1 = “urban,” 2 = “rural”). Adolescent income was operationalized as the amount of pocket money received from any household member. Academic performance was based on adolescents’ subjective evaluation of their academic achievement, rated on a scale from 1 (strongly dissatisfied) to 5 (strongly satisfied). Romantic relationship status was coded as a dummy variable (1 = “yes”, 0 = “no”).

### 3.5. Data Analysis

All analyses were conducted using SPSS 26.0. First, a correlation analysis was performed to provide an overall descriptive overview of the study variables. Next, hierarchical regression models were employed to examine the effects of control variables (gender, school location, monthly income, academic performance, and romantic relationship), the independent variables (online and offline communication), the moderator (age cohort), and their interaction terms on the quality of interpersonal relationships. Finally, the PROCESS macro (version 3.4; Hayes, 2013) was used to test the mediating role of offline communication in the relationship between online communication and the quality of interpersonal relationships. The indirect effect was estimated with Model 4 using 5000 bootstrap resamples and percentile 95% confidence intervals, and the standardized indirect effect size was also reported.

## 4. Results

### 4.1. Preliminary Analyses

Among the 1486 adolescent participants, female adolescents (M = 0.54, SD = 0.51) engaged in more offline communication than male adolescents (M = 0.48, SD = 0.50), t(1484) = 2.24, *p* < 0.05. Urban adolescents (M = 0.58, SD = 0.52) engaged in more offline communication than rural adolescents (M = 0.43, SD = 0.47), t(1462) = 5.67, *p* < 0.001. Adolescents in romantic relationships reported higher levels of both online communication (M = 3.88, SD = 0.63) and offline communication (M = 1.20, SD = 0.86) compared with those not in romantic relationships (M = 3.00, SD = 1.26 and M = 0.49, SD = 0.49, respectively).

As shown in Table 2, age cohort was positively correlated with online communication (r = 0.35, *p* < 0.001) and offline communication (r = 0.26, *p* < 0.001) but was not significantly associated with the quality of interpersonal relationships (r = 0.04, *p* > 0.05).

### 4.2. Hypothesis Testing

Hypothesis 1 posited that online communication is positively related to offline communication among adolescents. Consistent with this hypothesis, online communication was positively associated with offline communication (β = 0.16, t = 5.73, *p* < 0.001).

Hypothesis 2 posited that online communication is positively related to the quality of interpersonal relationships among adolescents. In line with this prediction, online communication was positively associated with the quality of interpersonal relationships (β = 0.07, t = 2.26, *p* < 0.05).

Hypothesis 3 posited that offline communication is positively related to the quality of interpersonal relationships among adolescents. Supporting this hypothesis, offline communication was positively associated with the quality of interpersonal relationships (β = 0.10, t = 3.43, *p* < 0.01).

Hypothesis 4 posited that offline communication mediates the relationship between online communication and the quality of interpersonal relationships. Mediation was tested with Model 4 of the PROCESS macro for SPSS. Based on 5000 bootstrap resamples and percentile 95% confidence intervals, the indirect effect of online communication on the quality of interpersonal relationships through offline communication was significant, with an indirect effect estimate of 0.11 (95% CI [0.004, 0.019]) and a standardized effect size of 0.17 (95% CI [0.007, 0.030]). Thus, Hypothesis 4 was supported.

**Figure 2:**
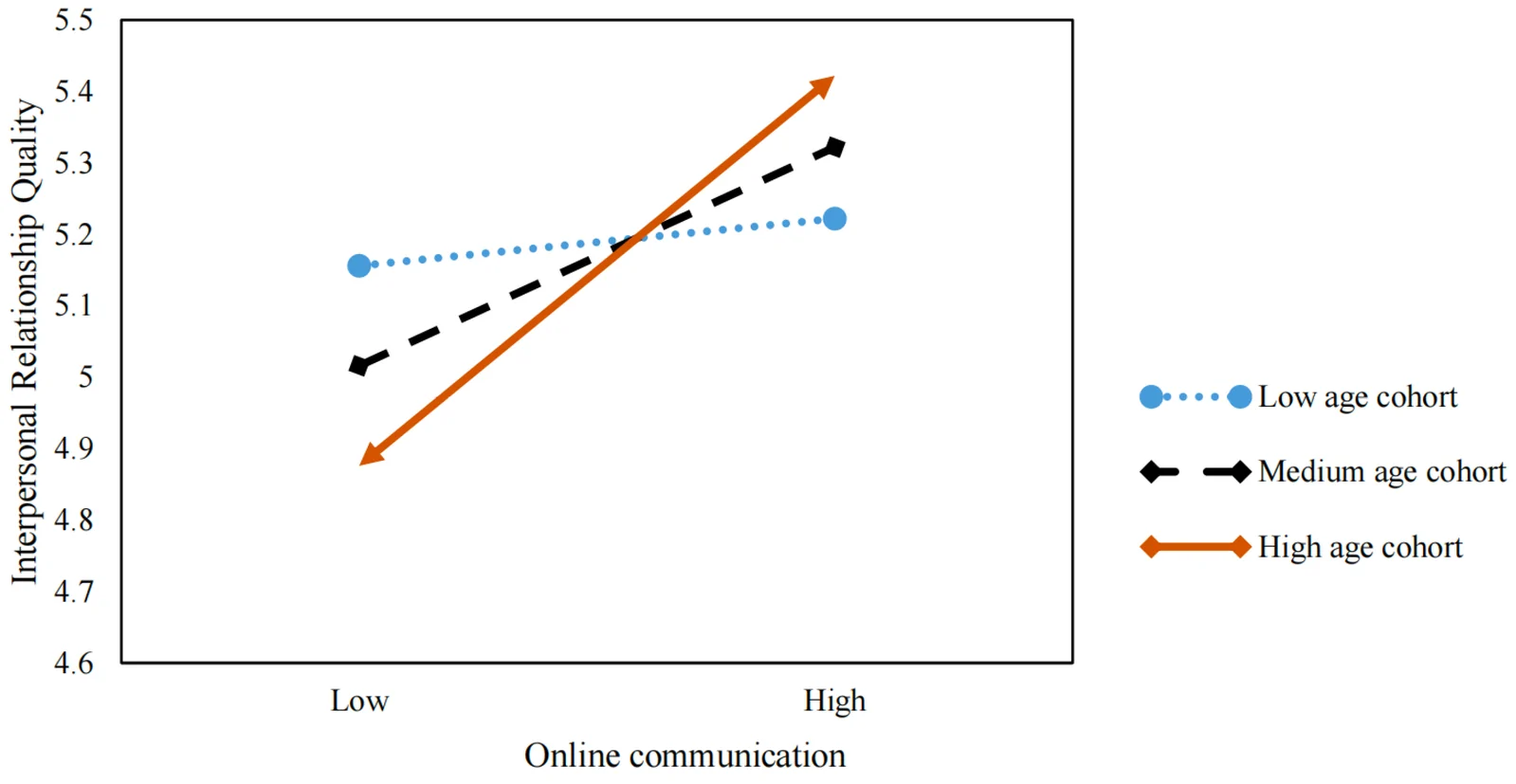
The interaction between the age cohort and online communication.

**Figure 3:**
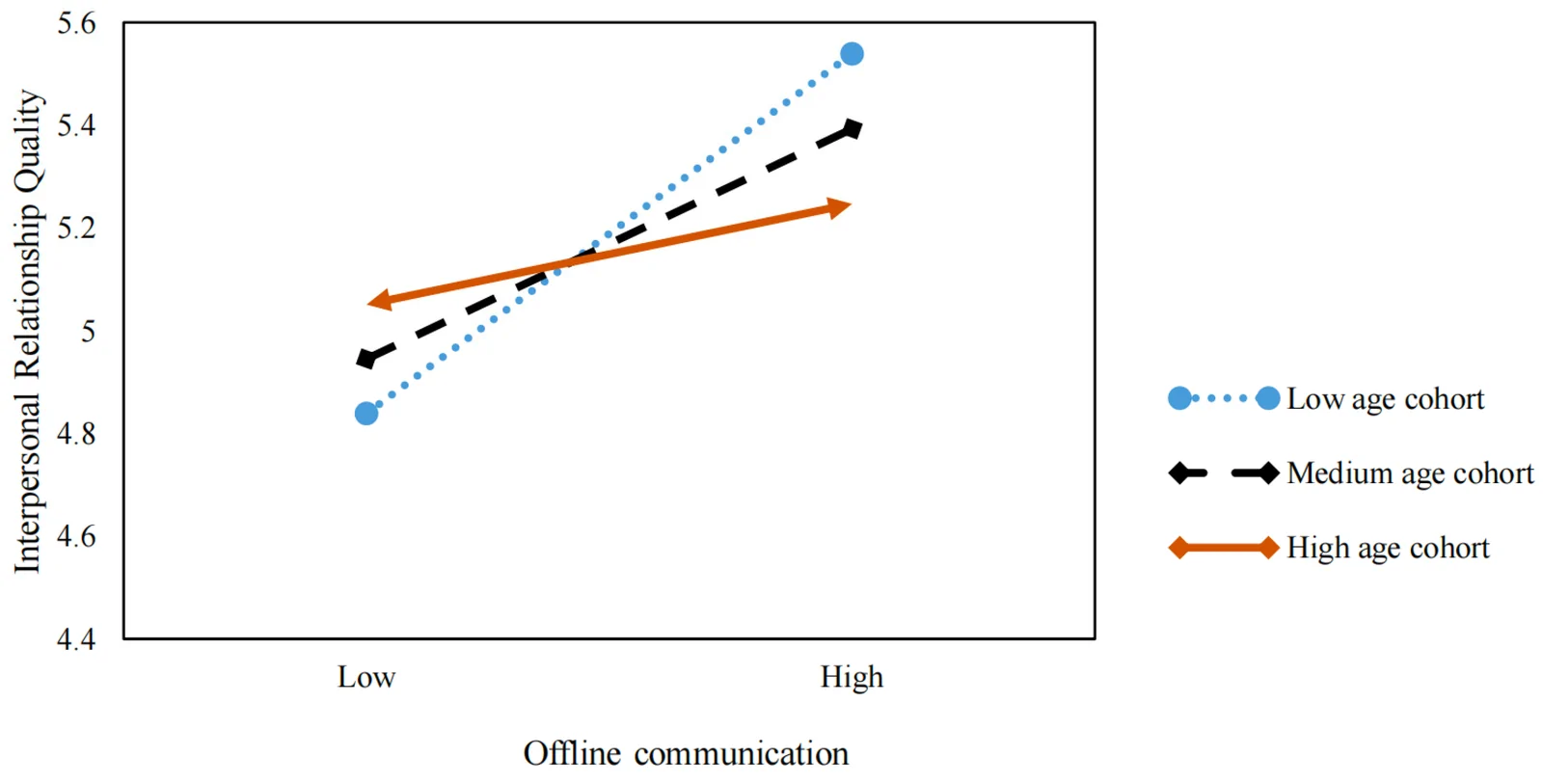
The interaction between the age cohort and offline communication.

Hypothesis 5a posited that older adolescents regard online communication as more important in predicting the quality of their interpersonal relationships than do younger adolescents. Hypothesis 5b posited that older adolescents regard offline communication as less important in predicting the quality of their interpersonal relationships than do younger adolescents. A hierarchical regression analysis was conducted to examine the moderating effect of age cohort on the association between online (offline) communication and the quality of interpersonal relationships, with the dependent variable centered (see Table 3). In the first step (Model 1), control variables including gender, school location, adolescent income, academic performance, and romantic relationship were entered into the regression equation. In the second step (Model 2), online communication was added. In the third step (Model 3), offline communication and age cohort were entered. In the final step (Model 4), the interaction terms of online communication × age cohort and offline communication × age cohort were added. As shown in Figure 2, the interaction between online communication and age cohort was significant (β = 0.06, t = 2.12, *p* < 0.05). As shown in Figure 3, the interaction between offline communication and age cohort was also significant (β = −0.07, t = −2.36, *p* < 0.05). Thus, both Hypotheses 5a and 5b were supported. Thus, both Hypotheses 5a and 5b were supported. According to Cohen’s (1992) guidelines, the changes in explained variance at each step (ΔR^2^ = 0.01) correspond to small effect sizes (f^2^ ≈ 0.01); although small, such increments are not trivial in a large, nationally representative sample, where even modest systematic associations may carry practical implications at the population level.

## 5. Discussion

The present study systematically examined the effects of online and offline communication on the quality of interpersonal relationships among Chinese adolescents. The results demonstrated that adolescents who engaged in more online communication also tended to participate in more offline communication. Furthermore, both online and offline communication jointly predicted the quality of interpersonal relationships among adolescents. Importantly, age cohort was found to moderate the effects of both online and offline communication on the quality of interpersonal relationships.

### 5.1. Online Communication and Offline Communication

Consistent with the Stimulation Hypothesis, the present study found that adolescents who engaged in more online communication also participated in more offline communication in terms of both the variety and intensity of social activities. In other words, Chinese adolescents’ online communication appears to be embedded in, rather than detached from, their offline social lives: adolescents who communicate more actively online are also those who more frequently interact with their friends face to face. This finding may be attributable to the fact that adolescents primarily interact with existing offline friends in online settings and use the internet mainly to strengthen these friendships. It is only under circumstances in which adolescents predominantly interact with strangers online that online communication tends to be associated with reduced time with offline friends and diminished offline communication. Previous literature has repeatedly documented a positive association between online-mediated interaction and offline social engagement. For instance, Valkenburg and Peter (2007b) observed that internet-based exchanges, including instant-messaging and chat activities, correlate with greater in-person time spent among peers. Such beneficial outcomes can be partly explained by intrinsic properties of online communication, notably perceived controllability and reduced social cues. A body of empirical work illustrates that these attributes can promote intimate self-disclosure (Joinson, 2001; Leung, 2002; McKenna et al., 2002; Tidwell & Walther, 2002; Valkenburg & Peter, 2007a), a well-established precursor of mutual interpersonal attraction (N. L. Collins & Miller, 1994). It follows, therefore, that internet-enabled intimate self-disclosure may help explain possible improvements in the quality of adolescent friendships.

Other scholars have argued that social media may serve as a substitute for face-to-face interaction, potentially leading to deteriorating relationship quality and decreased intimacy among users, as online communication may render adolescents less interested in face-to-face communication with their friends (Rosen, 2007). The Displacement Hypothesis predicted negative associations between time spent in online communication and time with parents, although time with friends was not displaced (Zhuo et al., 2025). The present study posits that online communication does not function as a substitute for face-to-face interaction; rather, it serves as an extension of communication with existing face-to-face partners. Collectively, these findings call for further investigation into the extent to which online communication substitutes for or complements offline communication. More specifically, future research should examine the specific aspects and types of interpersonal relationships, the characteristics of online communication, and the social needs of adolescents for which online communication may substitute offline communication.

### 5.2. Online as Well as Offline Communication and the Quality of Interpersonal Relationships

The present study found that both online and offline communication are positively associated with the quality of interpersonal relationships. Notably, offline communication demonstrated a stronger association with the quality of interpersonal relationships among adolescents. This pattern suggests that, despite Chinese adolescents’ extensive engagement with digital media, face-to-face interaction remains the most salient source of relational closeness during adolescence, whereas online communication serves a complementary rather than a substitutive role. This finding is consistent with previous research indicating that internet use for social interaction purposes can enhance the quality of interpersonal relationships. For example, Valkenburg and Peter (2007a) proposed an indirect relationship between online communication and friendship quality, mediated by time spent with existing friends. They suggested that individuals with higher social anxiety are more motivated to use blogs to form new friendships, as the blogosphere makes highly anxious individuals feel more comfortable interacting with others. Similarly, Tian (2013) found that more motivated bloggers made more new friends and reported higher quality in these new friendships.

Research has also shown that instant messaging was predominantly used to communicate with existing friends, whereas chatting in public chatrooms was relatively more often used to interact with strangers (Mýlek et al., 2024). Accordingly, instant-messaging activities correlate positively with multiple dimensions of romantic-relationship quality and close-friendship quality. By contrast, chat-room participation shows negative links to the quality of best-friend bonds (Blais et al., 2008; Mýlek et al., 2024). Other researchers have explored the process of online friendship formation. For instance, Peter et al. (2005) identified introversion/extraversion, online self-disclosure, the motive for social compensation, and frequency of online communication as predictors of adolescent friendship formation. Furthermore, several scholars have linked online communication, friendship quality, and well-being in an integrated framework. There is broad agreement that friendship quality is an important predictor of well-being (Baiocco et al., 2011; Hartup & Stevens, 1997; Mýlek et al., 2024). Taken together with the existing literature, the present study underscores the need to simultaneously compare the effects of online and offline communication on friendship quality, rather than examining only the indirect effect of online communication on friendship quality through offline communication.

### 5.3. The Moderating Effect of Age Cohort

The significant moderating effect of age cohort highlights that the role of online communication in adolescents’ interpersonal relationships varies across different developmental stages. Specifically, the results indicate that older adolescents place greater emphasis on online communication in their interpersonal relationships relative to offline communication. This finding is consistent with research demonstrating that online communication and closeness to friends increase with age (Angelini et al., 2026). More specifically, age is positively associated with time spent on instant messaging (Mýlek et al., 2024). Instant messaging’s dyadic, real-time, and private affordances make it well-suited for sustaining intimate interactions among close friends. For adolescents embedded in high-quality offline friendships, these same affordances may also support relatively effortless expansion of online social networks, as social competencies nurtured within strong pre-existing bonds can translate to forming new digital connections (Anderson, 2001). As prior research has observed, intensified individualization and growing physical distance from family and peers within industrialized societies limit opportunities for face-to-face peer engagement. Against this backdrop, instant messaging operates as a vital compensatory channel that mitigates constraints imposed by geographic separation (Wolak et al., 2003). When considered alongside one another, these documented properties of digitally mediated communication help explain why such platforms may hold particular appeal for individuals who rate themselves low in social competence.

### 5.4. Practical Implications

The present study indicates that age cohort is positively associated with both online and offline communication, suggesting that parents and educational practitioners should attend to adolescents’ psychosocial developmental needs. Adolescents may pursue the overarching goal of building meaningful relationships in both offline and online communication contexts. Notably, offline communication remains the strongest predictor of friendship quality among adolescents, although this effect diminishes as adolescents grow older. Stakeholders may therefore educate and assist younger adolescents in building and maintaining friendships in offline contexts, particularly for adolescents experiencing social anxiety (Bonetti et al., 2010; Tian, 2013). For example, schools and community programs could design age-tailored social skills training that combines offline group activities with guided online communication practice, helping younger adolescents build friendships in face-to-face settings while gradually learning to maintain them online.

Second, the present study indicates that higher levels of online communication are also associated with better friendship quality, and this effect intensifies with age. On the one hand, online communication provides opportunities that may facilitate adolescents’ psychosocial development, particularly in the domains of identity and intimacy. On the other hand, research has shown that online communication may reinforce peer communication at the expense of communication with parents, who may lack sufficient knowledge about their children’s online activities (Blahošová et al., 2025). Parents should therefore consider how to interact effectively with their children regarding internet use and mitigate potential negative outcomes such as addiction, cyberbullying, sexual exploration, and unwanted sexual solicitation. Moreover, given the reciprocal effects between internet addiction and approaches to friendship, internet addiction and preference for online social interaction condition young people’s tendency to seek friendships from individuals met online (Smahel et al., 2012). Stakeholders should guide adolescents toward healthy and constructive use of the internet and social media. In particular, given that the benefits of online communication depend on how it is used, media literacy education should encourage active, relationship-oriented use rather than prolonged passive consumption (Seidman et al., 2025). Parents are encouraged to communicate openly with their children about their online experiences and to jointly establish reasonable rules for internet use, thereby balancing adolescents’ relational needs with online safety.

### 5.5. Limitations and Future Directions

Although the present study tested the proposed hypotheses using a nationally representative survey, several limitations should be noted when interpreting the findings. First, because the study relied on secondary data, some variables were recoded to align with the measures employed in the present study. Notably, the dependent variable, quality of interpersonal relationships, was assessed using a single item. Future studies should employ validated scales of adolescent interpersonal relationships (e.g., Garthoeffner et al., 1993) to examine the effects of online communication on overall relationship quality or specific types of interpersonal relationships such as parent–child and teacher–student relationships.

Second, two measurement and design issues should be noted. Although the CFPS is a longitudinal panel, the present analyses relied on cross-sectional data from a single wave. Therefore, the observed associations cannot support causal inferences, and future research should exploit the longitudinal structure of the database to examine the temporal dynamics among online communication, offline communication, and relationship quality. In addition, offline communication was indexed by the frequency and variety of shared social activities (e.g., going out for dinner or karaoke), which captures activity-based face-to-face interaction rather than the content or depth of communication per se. Future studies should complement such indicators with direct measures of communication. Moreover, the online communication measure averaged a frequency item and an importance item and therefore cannot distinguish between active and passive forms of online communication, which may differentially relate to adolescents’ relationships and well-being. Future research should employ multi-item scales that differentiate these modes.

Third, the present study examined only the moderating role of age cohort in the association between online (offline) communication and the quality of interpersonal relationships. Future research should investigate additional moderators, such as personality traits, academic performance, and family and school environment (Kraut et al., 1998; Angelini et al., 2026). Furthermore, there may be mediating mechanisms linking online (offline) communication to the quality of interpersonal relationships. Scholars could extend the present study by exploring different pathways between online (offline) communication and interpersonal relationships among adolescents.

Fourth, because the participants in the present study were adolescents between the ages of 10 and 15, we identified linear moderating effects of age cohort on the relationship between online (offline) communication and the quality of interpersonal relationships. However, there may also be curvilinear effects of age cohort (Pollet et al., 2011). Future studies should include adolescents above 15 years of age to further examine this possibility.

## 6. Conclusions

The present study integrated the Displacement Hypothesis and the Stimulation Hypothesis by directly comparing the effects of online and offline communication on the quality of interpersonal relationships among Chinese adolescents. The findings revealed that offline communication mediates the relationship between online communication and the quality of interpersonal relationships. Furthermore, age cohort moderates the effects of both online and offline communication on the quality of interpersonal relationships. These findings advance our understanding of the effects of online communication on adolescent development and highlight the importance of considering developmental stage when examining the interplay between online and offline social interactions. Given the cross-sectional design of the present study, these findings should be interpreted as associations rather than causal effects, and longitudinal evidence is needed to confirm the developmental dynamics proposed here.

## Figures and Tables

**Table 1:** Demographics and controlled variables of the overall sample.

	N	Mean (Percentage)	SD
*Age*		12.85	1.61
*Gender*			0.50
Female	677	45.6%	
Male	809	54.4%	
*School location*			0.50
Urban	699	47.7%	
Rural	765	52.3%	
*Adolescent’s income*		80.59	134.54
*Academic performance*		3.13	0.81
*Romantic relationship*			0.49
Yes	23	1.5%	
No	1463	98.5%	

**Table 2:** Bivariate correlations of study variables.

Variable	1	2	3	4	5	6	7	8	9
1. Gender	_								
2. School location	−0.03	_							
3. Adolescent’s income	0.00	−0.13 ***	_						
4. Academic performance	−0.03	−0.03	−0.01	_					
5. Romantic relationship	0.01	0.03	−0.06 *	−0.01	_				
6. Age cohort	−0.01	−0.21 ***	0.26 ***	−0.10 ***	−0.09 **	_			
7. Online communication	0.01	−0.05	0.17 ***	−0.04	−0.09 **	0.35 ***	_		
8. Offline communication	−0.06 *	−0.15 ***	0.24 ***	−0.01	−0.17 ***	0.26 ***	0.21 ***	_	
9. Quality of interpersonal relationships	0.03	−0.05	0.07 *	0.24 ***	−0.04	0.04	0.08 **	0.13 ***	_

*Note*. *** *p* < 0.001, ** *p* < 0.01, * *p* < 0.05.

**Table 3:** The hierarchical regression of online communication as well as offline communication on the quality of interpersonal relationships.

Predictor Variable	Standardized Regression Coefficients			
	Model 1	Model 2	Model 3	Model 4
Gender	0.04			
School location	−0.01			
Adolescent’s income	0.09 **			
Academic performance	0.25 ***			
Romantic relationship	−0.04			
Adjusted R^2^	0.07			
Online communication		0.07 *		
R^2^		0.08 ***		
∆R^2^		0.01 ***		
Offline communication			0.10 **	
Age cohort			0.01	
R^2^			0.09 ***	
∆R^2^			0.01 ***	
Online communication × Age cohort				0.06 *
Offline communication × Age cohort				−0.07 *
R^2^				0.10 ***
∆R^2^				0.01 ***

*Note*. *** *p* < 0.001, ** *p* < 0.01, * *p* < 0.05.

## Data Availability

Data supporting reported results can be found in the publicly archived datasets at: https://cfpsdata.pku.edu.cn/#/home (this website requires registration for download and use).
